# Methylene Blue Dye Adsorption on Iron Oxide-Hydrochar Composite Synthesized via a Facile Microwave-Assisted Hydrothermal Carbonization of Pomegranate Peels’ Waste

**DOI:** 10.3390/molecules28114526

**Published:** 2023-06-02

**Authors:** Manal Hessien

**Affiliations:** Department of Chemistry, College of Science, King Faisal University, P.O. Box 400, Alahsa 31982, Saudi Arabia; mhessien@kfu.edu.sa

**Keywords:** microwave-assisted hydrothermal carbonization method, pomegranate peels, iron oxide-hydrochar composites, methylene blue dye, adsorption

## Abstract

The toxicity of dyes has a long-lasting negative impact on aquatic life. Adsorption is an inexpensive, simple, and straightforward technique for eliminating pollutants. One of the challenges facing adsorption is that it is hard to collect the adsorbents after the adsorption. Adding a magnetic property to the adsorbents makes it easier to collect the adsorbents. The current work reports the synthesis of an iron oxide-hydrochar composite (FHC) and an iron oxide-activated hydrochar composite (FAC) through the microwave-assisted hydrothermal carbonization (MHC) technique, which is known as a timesaving and energy-efficient method. The synthesized composites were characterized using various techniques, such as FT-IR, XRD, SEM, TEM, and N_2_ isotherm. The prepared composites were applied in the adsorption of cationic methylene blue dye (MB). The composites were formed of crystalline iron oxide and amorphous hydrochar, with a porous structure for the hydrochar and a rod-like structure for the iron oxide. The pH of the point of zero charge (pHpzc) of the iron oxide-hydrochar composite and the iron oxide-activated hydrochar composite were 5.3 and 5.6, respectively. Approximately 556 mg and 50 mg of MB dye was adsorbed on the surface of 1 g of the FHC and FAC, respectively, according to the maximum adsorption capacity calculated using the Langmuir model.

## 1. Introduction

The incessant population growth, along with the industrial civilization, has resulted in a high negative impact on the environment. Water contamination is highly affected by the tremendous amounts of industrial waste. Diverse sectors of industries, such as textile, plastics, and cosmetics, discharge synthetic dyes into water streams. Due to the poisonous and cancer-causing nature of these dyes, continuous dye discharge into the environment is a highly significant environmental issue. Additionally, the accumulation of dyes in aquatic systems decreases the light dispersion and photosynthesis. Furthermore, because of its biotransformation products, it can seriously harm aquatic life [1]. Therefore, before releasing wastewater into water bodies, the colors must be removed from it. For the purpose of extracting dyes from wastewater, several physical, biological, electrochemical, and chemical techniques have been developed.

One of the more alluring methods for eliminating organic dyes from water systems is adsorption. This is primarily because it is a straightforward, highly effective, and inexpensive method that requires little maintenance and is available with a very large selection of adsorbents. Numerous researchers have successfully removed water contaminants through adsorption using a variety of adsorbents, including ZnO [2], ZnO/biochar [3], hydrochar [4,5,6], clay [7], CuO-ZnO-C nanocomposites [8], magnetic hydrochar [9], ZnO-NiO nanocomposites [10], magnetic biochar [11], iron nanoparticles [12], biochar [13,14], and NiO nanoparticles [15].

Despite all the advantages of adsorption, there is still one challenge regarding the ease of collecting the adsorbents after the adsorption process is completed. One way to solve this issue is adding magnetic properties to the hydrochar, which can be added in a consecutive way or a simultaneous way [9]. In the consecutive method, hydrochar is prepared, then the magnetic material is added, which may be a metal or a metal oxide. This method sometimes results in blocking the pores, and the magnetic materials can be leached out [9,16]. In the simultaneous way, the magnetic precursor is added to the hydrochar during the synthesis [17]. In this method, the magnetic material is not only on the hydrochar surface, but it is well-dispersed through the hydrochar [18,19].

There are several ways to transfer biowaste into useful carbon-based materials. Biochar is prepared through pyrolysis, which is a thermal treatment in the absence of oxygen [14]. Hydrochar is prepared through a hydrothermal treatment, and it is more commonly known as hydrothermal carbonization [20,21,22,23]. Activated hydrochar is prepared in the same way as the hydrochar, but in the presence of activating agents such as NaOH, ZnCl_2_, and citric acid [23,24]. Recently, a microwave is used as a heating source instead of normal heating sources, and this treatment is called the microwave-assisted hydrothermal carbonization (MHC) method [24,25,26,27,28]. The microwave-assisted hydrothermal carbonization (MHC) method offers several advantages as it is a greener method and saves time and energy [25,29,30]. 

M. Hessien applied MHC treatment on pomegranate peels and reported that the prepared hydrochar was rich with oxygenated functional groups, which facilitate the adsorption of MB dye to attain a maximum adsorption capacity of 196 mg/g [6]. A. Patino et al. prepared iron oxide-hydrochar through a two-step process. First, the hydrothermal carbonization of sunflower husks was used to prepare the hydrochar. Second, the iron hydroxide was coprecipitated on the hydrochar, then subjected to hydrothermal treatment. The measured surface area was 55 m^2^/g and the average pore diameter was 10 nm [9]. M. Kim et al. prepared iron oxide-hydrochar from sugarcane bagasse through hydrothermal carbonization, followed by a modification with iron oxide [16]. O. Kazak et al. prepared a magnetic hydrochar through a co-hydrothermal treatment with vinasse as the source for the hydrochar and red mud as the source for iron oxide. Their sample had a surface area of 23 m^2^/g, a pore size 1.4–5 nm, and a pore volume of 0.071 cm^3^/g, and the sample was applied in the removal of Pb ions [31]. 

To the best of the author’s knowledge, this work is the first to study the simultaneous formation of iron oxide-hydrochar through microwave-assisted hydrothermal carbonization (MHC) treatment. This work has the objectives of using pomegranate peels as a feedstock and applying MHC treatment in the presence of an iron oxide precursor to prepare an iron oxide-hydrochar composite (FHC); to study the effect of ZnCl_2_ as an activating agent on pomegranate peels and an iron oxide precursor to prepare an iron oxide-activated hydrochar composite (FAC); and to compare the adsorption efficiency of methylene blue (MB) on the prepared composites.

## 2. Results and Discussion

### 2.1. FT-IR Characterization of Prepared Samples

**Figure 1:**
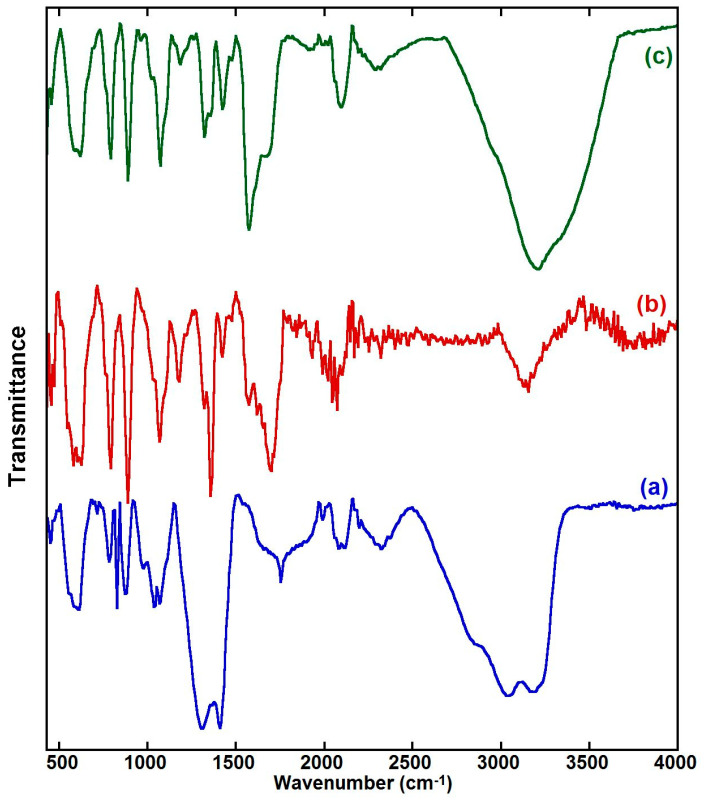
FT-IR spectra of (a) iron oxide (F), (b) iron oxide-hydrochar composite (FHC), and (c) iron oxide-activated hydrochar composite (FAC).

Figure 1 shows: (a) the FT-IR spectra of the iron oxide (F); (b) the iron oxide-hydrochar composite (FHC); and (c) the iron oxide-activated hydrochar composite (FAC), which were synthesized using the microwave-assisted hydrothermal carbonization technique. The formation of iron oxide is confirmed in the three samples as the three spectra show vibrations at about 457 cm^−1^ and 550 cm^−1^, which may be referred to as the tetrahedral and octahedral Fe-O of magnetite. Moreover, the vibrations at 610 cm^−1^ and 880 cm^−1^ may be referred to as maghemite and goethite, respectively [32,33]. 

Figure 1b shows peaks at 1077 cm^−1^ for aromatic CH, at 1360 cm^−1^ for quinonic OH, and at 1575 cm^−1^ for C=C; the peaks at 2070 cm^−1^ are assigned for allene groups of aromatic rings, and at 3150 cm^−1^, for aromatic CH. Moreover, the oxygenated functional groups are observed with a peak at 1427 cm^−1^ for –COOH, at 1710 cm^−1^ for -CO, and at 3750 cm^−1^ for –OH groups. The FT-IR confirms the transformation of the pomegranate peels into hydrochar and the formation of iron oxide. The activated hydrochar formation is established through the presence of the following peaks, as shown in Figure 1c: the peaks at 1077 cm^−1^, at 1185 cm^−1^, and at 3150 cm^−1^ are assigned for aromatic CH; at 1360 cm^−1^ for quinonic OH, at 1575 cm^−1^ for C=C, and at 2100 cm^−1^ for allene groups of aromatic rings. Moreover, the surface functional groups are observed with a peak at 1427 cm^−1^ for –COOH, at 1675 cm^−1^ for –C=O, and a broad band at 2700–3600 cm^−1^ for –OH groups [5,14]. The FT-IR confirms the transformation of pomegranate peels into activated hydrochar and the formation of iron oxide.

It is worth mentioning that the carbonyl group has been observed at 1710 cm^−1^ in the FHC sample and at 1675 cm^−1^ in the FAC sample. In our previous work [6], the carbonyl group was observed at 1700 cm^−1^ in the hydrochar sample and at ~1690–1725 cm^−1^ for the activated hydrochar sample. In addition, the allene peak was observed in the samples FHC and FAC. However, the allene peak was not observed in the hydrochar sample in the previous work and was observed in activated hydrochar sample only.

These differences may indicate that the iron oxide precursors (FeCl_3_ and FeCl_2_) acted as activating agents, as ZnCl_2_ had acted in the previous work [6].

### 2.2. XRD Characterization of Prepared Samples

**Figure 2:**
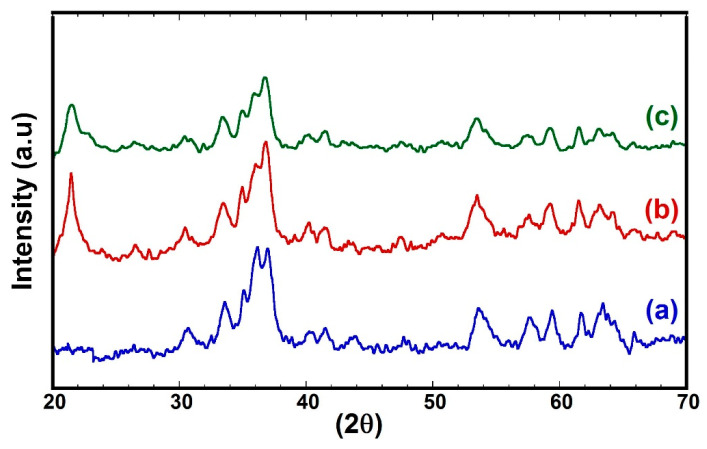
XRD of (a) iron oxide (F), (b) iron oxide-hydrochar composite (FHC), and (c) iron oxide-activated hydrochar composite (FAC).

Figure 2 represents the XRD diffractograms of: (a) the iron oxide; (b) the iron oxide-hydrochar composite; and (c) the iron oxide-activated hydrochar composite prepared through MHC treatment. In Figure 2a, the peaks at 2Ɵ = 30.70°, 36.2°, 41.9°, 53.29°, 57.47°, and 61.65° can be ascribed to the crystalline planes (200), (311), (400), (422), (511), and (440) of magnetite, according to JCPDS file no: 00-003-0863. Magnetite is an inverse spinel structure with a face centered cubic lattice [9,32]. The diffraction peaks at 2Ɵ = 33.46°, 35.11°, 36.81°, 40.30°, and 53.29° can be assigned to (301), (201), (111), and (212), respectively, of goethite, according to JCPDS file no: 29-0713. Goethite is an orthorhombic structure of α-FeOOH [34].

Figure 2b represents the XRD diffractogram of the iron oxide-hydrochar composite synthesized through MHC treatment. There is a peak at about 2Ɵ = 22°, which refers to amorphous carbon, in addition to the other peaks reported in the iron oxide sample [35]. Figure 2c represents the XRD diffractogram of the iron oxide-activated hydrochar composite synthesized through MHC treatment. There is a peak at about 2Ɵ = 22°, which refers, in this case, to activated hydrochar, in addition to the observed peaks in the iron oxide sample [9,16]. The XRD characterization confirms the formation of amorphous carbon and crystalline iron oxide.

### 2.3. Morphology of Prepared Samples

**Figure 3:**
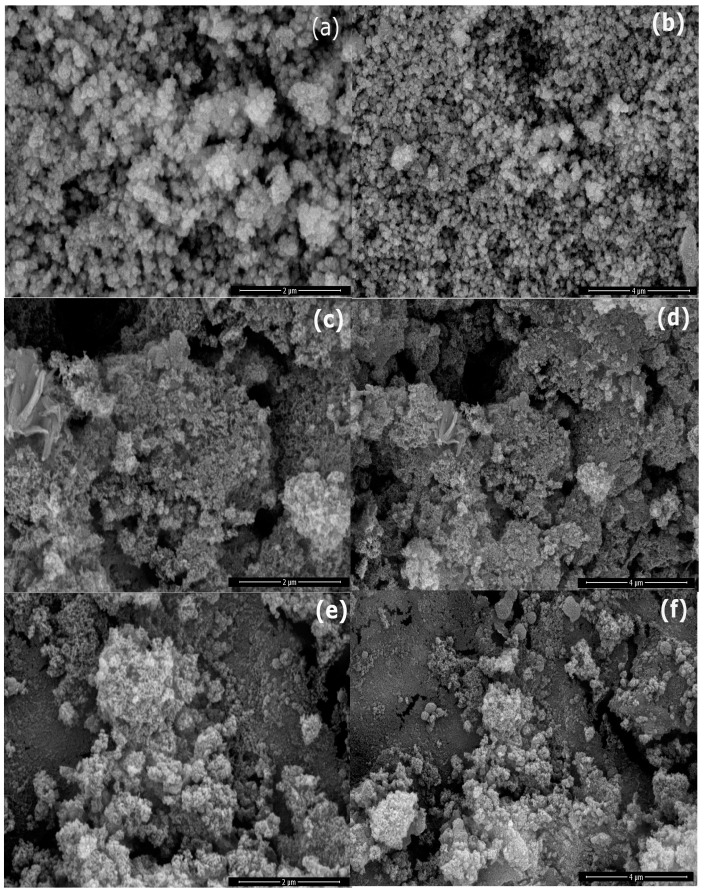
SEM images of (**a**,**b**) iron oxide, (**c**,**d**) iron oxide-hydrochar composite, (**e**,**f**) iron oxide-activated hydrochar composite.

Figure 3 represents the SEM images of the iron oxide, iron oxide-hydrochar composite, and iron oxide-activated hydrochar composite. The iron oxide is formed of agglomerated spherical particles, as shown in Figure 3a,b. The iron oxide-hydrochar composite and iron oxide-activated hydrochar composite show a porous microstructure with a rough surface [36].

**Figure 4:**
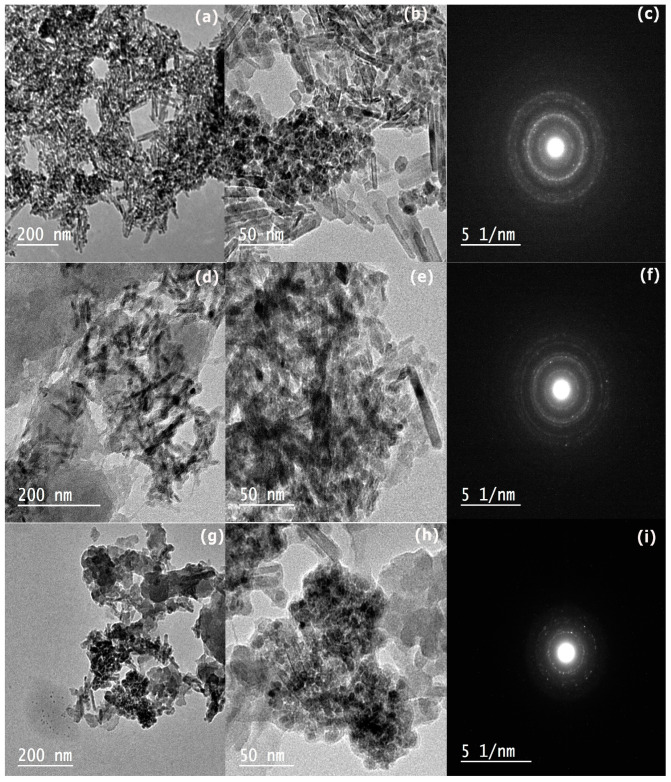
TEM images of (**a**–**c**) iron oxide, (**d**–**f**) iron oxide-hydrochar composites, (**g**–**i**) iron oxide-activated hydrochar composite.

Figure 4 shows the TEM images of the iron oxide, iron oxide-hydrochar composite, and iron oxide-activated hydrochar composite. The iron oxide has a rod-like structure with 4–10 nm width and a strict SAED ring, confirming the crystallinity of the formed iron oxide, as shown in Figure 4a–c. Figure 4d,e show that the TEM of the iron oxide-hydrochar composite with iron oxide has a rod-like structure and a porous structure for hydrochar. Figure 4f represents the SAED ring, which confirms the crystallinity structure of the iron oxide. However, the rings are less sharp than the pure iron oxide because of the presence of amorphous hydrochar. Figure 4g,h show the TEM images of the iron oxide-activated hydrochar composite, which show the iron oxide to have a rod-like structure with a porous open microstructure for activated hydrochar. Figure 4i shows the SAED ring, which is less sharp than the pure iron oxide because of the presence of amorphous activated hydrochar.

### 2.4. The pH of Point of Zero Charge (pHpzc) 

**Figure 5:**
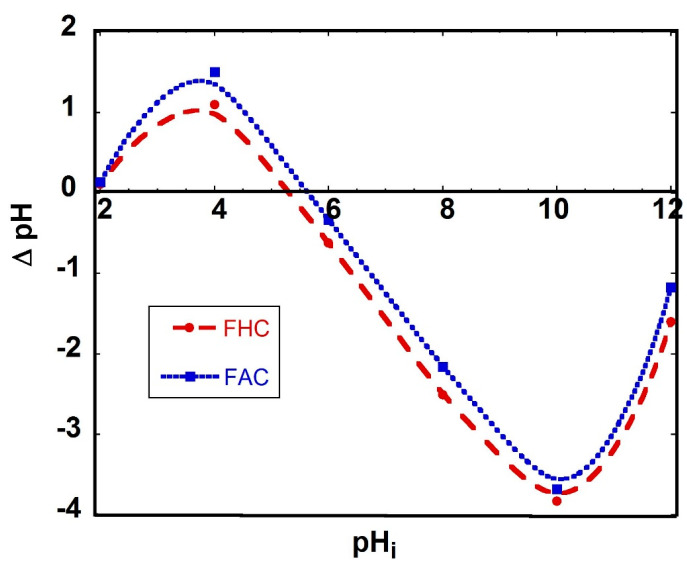
pH of point of zero charge of FHC and FAC.

Figure 5 shows the initial pH on the X-axis and ∆pH on the Y-axis. The intersection between the curve and the x-axis is the pH of the point of zero charge (PZC). The pH of the PZC of the iron oxide-hydrochar composite and iron oxide-activated hydrochar are 5.3 and 5.6, respectively. The pH of the PZC represents the pH at which the surface charge of the adsorbent is zero. If the pH of a solution is lower than the pH of the PZC, the surface charge is positive, and vice versa. The adsorption experiment in this work was executed at pH 8. This pH was chosen to avoid the electrostatic repulsion between the cationic MB dye and the positive surface of the FHC and FAC at a pH less than 5 [37,38]. Moreover, it is recommended to carry out the adsorption of cationic dyes at a higher pH than the pH of the point of zero charge (PZC) of adsorbents in order to reduce the competition between protons and cationic dyes [39].

### 2.5. Adsorption Equilibrium of Methylene Blue Dye

**Figure 6:**
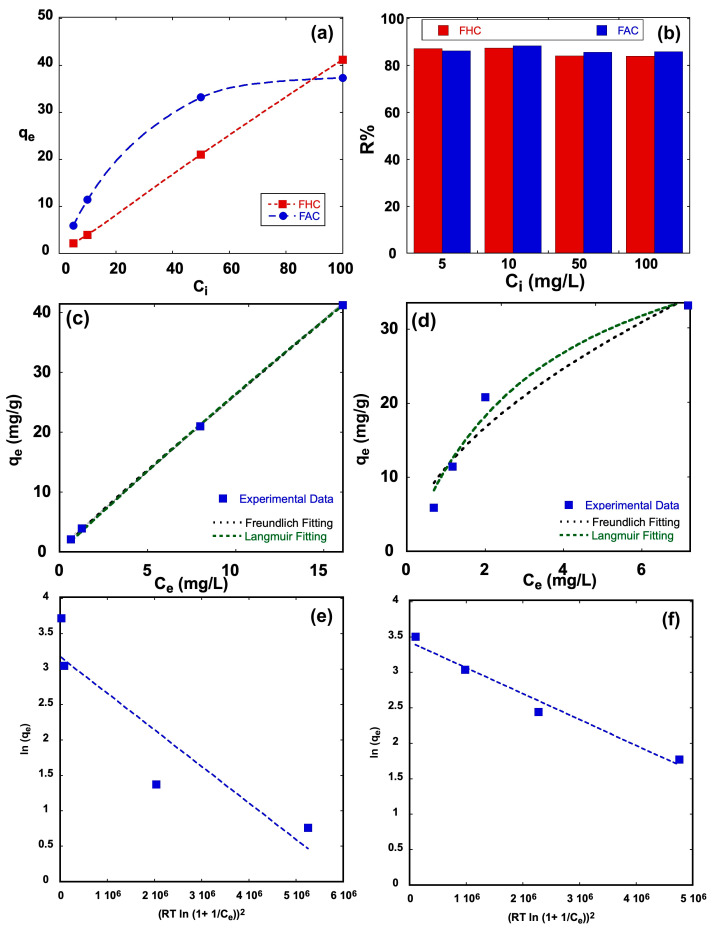
The relationship between (**a**) equilibrium adsorption capacity and initial MB dye concentration, (**b**) removal efficiency and initial MB dye concentration, (**c**) FHC experimental results and fitting models, (**d**) FAC experimental results and fitting models, (**e**) FHC Dubinin–Radushkevich fitting model, and (**f**) FAC Dubinin–Radushkevich fitting model.

Figure 6a represents the impact of the MB dye’s starting concentration (*C_i_*) on the equilibrium adsorption capacity (*q_e_*) after 1 day. When the starting concentration of the MB dye was 5 mg/L, the *q_e_* was 2.13 mg/g and 5.88 mg/g for the iron oxide-hydrochar composite and iron oxide-activated hydrochar, respectively. Increasing the starting concentration of the MB dye to 10 mg/L results in a corresponding increase in the *q_e_* to 3.94 mg/g and 11.46 mg/g for the iron oxide-hydrochar composite and iron oxide-activated hydrochar, respectively. A further increase in the starting concentration of the MB dye to 50 mg/L results in a corresponding increase in the *q_e_* to 21.00 mg/g and 33.22 mg/g for the iron oxide-hydrochar composite and iron oxide-activated hydrochar, respectively. When the starting concentration of the MB dye reaches 100 mg/L, the *q_e_* increases to 41.19 mg/g and 37.30 mg/g. The FAC sample shows a higher adsorption capacity than the FHC up to the initial concentration of 50 mg/L. The equilibrium adsorption capacity of the FHC sample shows a linear relationship with the initial concentration, suggesting that the surface of the FHC sample did not obtain the full amount of MB dye molecules. On the other hand, the equilibrium adsorption capacity of the FAC sample increases linearly, then has a plateau. The effect of the increase in the starting concentration of the Methylene Blue dye (*C_i_*) on the efficiency of removing MB (R%) is shown in Figure 6b. For the FHC sample, the R% values were 87.2%, 87.45%, 84%, and 83.9%, while the R% values of the FAC sample were 86%, 88%, 85.6%, and 85.5% when the starting concentrations of the Methylene Blue dye were 5, 10, 50, and 100 mg/L, respectively. 

The blue squares in Figure 6c show the measured results of the equilibrium adsorption capacity of the iron oxide-hydrochar composite, the dashed green line represents the Langmuir fitting with correlation coefficient R^2^ = 0.999, and the black dashed line represents the Freundlich fitting with correlation coefficient R^2^ = 0.999. The adsorption of methylene blue on FHC can be fitted with both models. The blue squares in Figure 6d show the measured results of the equilibrium adsorption capacity of the iron oxide-activated hydrochar composite, the dashed green line represents the Langmuir fitting with correlation coefficient R^2^ = 0.968, and the black dashed line represents the Freundlich fitting with correlation coefficient R^2^ = 0.93. The adsorption of methylene blue on FAC can be fitted better with the Langmuir model. The Langmuir model indicates that the adsorption of MB on FHC and FAC is that of monolayer adsorption on a homogenous surface [40]. The Freundlich fitting indicates that the surfaces of FHC and FAC may be heterogenous surfaces with nonuniform affinities and multilayer adsorption of MB dye on composites [41]. 

The parameters of these two models are represented in Table 1 and Table 2. The calculated maximum adsorption capacity (*q_L_*) is 556.33 mg/g for FHC, which is higher than the recorded value for FAC—50.42 mg/g. The interaction between the MB and the adsorbent can be evaluated via the Langmuir constant (*K_L_*). The *K_L_* values are 0.005 and 0.281 for FHC and FAC, respectively, which suggest a greater interaction between the molecules of the MB dye and the surface of the FAC composite. When the separation factor (*R_L_*) value is between 0–1, this indicates that the adsorption process is favorable. The separation factor *R_L_*, as seen in Table 2, drops from 0.976 to 0.668 for the FHC sample, and drops from 0.4155 to 0.0343 for the FAC sample, when the starting concentration rises from 5 ppm to 100 ppm. All of the values indicate favorable adsorption. From the Freundlich fit, the relative adsorption capacity *K_f_* is 2.99 and 11.27, the sorption intensity (*n_fr_*) is 1.059 and 1.781, and the parameter 1/*n_fr_* is 0.944 and 0.561 for the FHC and FAC samples, respectively. When the parameter 1/*n_fr_* is < 1, the surface is more heterogonous, and when it is close to 1, the surface is more homogenous [42]. It seems that the FHC sample is more homogenous than the FAC sample. 

**Figure 7:**
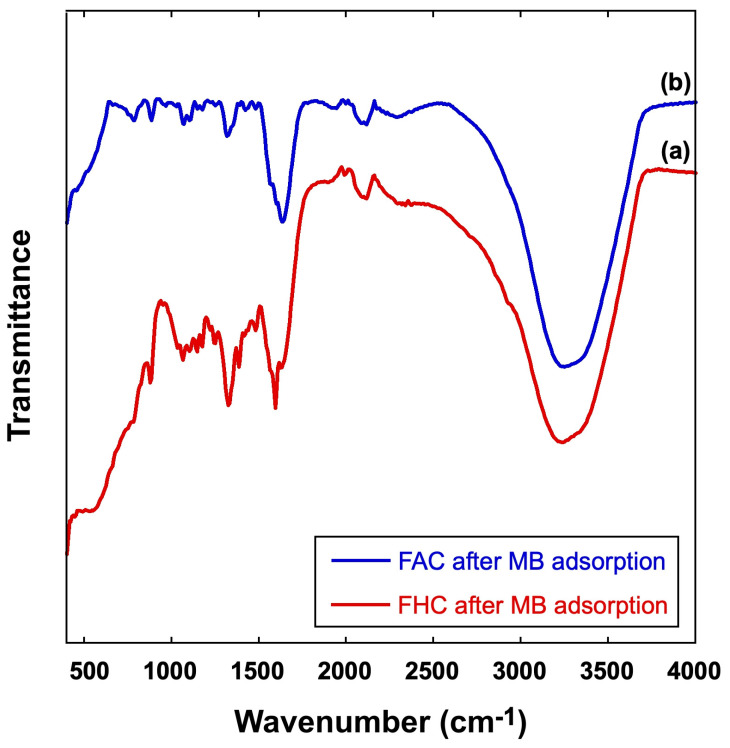
FT-IR spectra of (a) iron oxide-hydrochar composite (FHC) after MB adsorption and (b) iron oxide-activated hydrochar composite (FAC) after MB adsorption.

The fitting of the Dubinin–Radushkevich model is represented by the dotted line in Figure 6e,f for the FHC and FAC, respectively. The slope of the fitting model equals β, which is used to calculate the adsorption energy (E), which provides an indication about the nature of the adsorption process (i.e., chemical or physical). If E is less than 8 KJ/mol, it is considered to be a physical adsorption, and if E is 8–16 KJ/mol, it is considered to be a chemical adsorption [43,44,45,46,47]. Both samples show that their adsorption is most likely to be a chemical adsorption. FT-IR was performed for the FHC and FAC after the adsorption, and the FT-IR spectra are shown in Figure 7. When comparing the FT-IR of FHC and FAC before adsorption, as shown in Figure 1, with FT-IR of FHC and FAC after adsorption, as shown in Figure 7, it can be observed that the peaks of the OH, CO, and quinonic OH groups have been shifted to lower frequencies. Therefore, the surface functional groups are involved in the adsorption of MB dyes through hydrogen bond interactions. Moreover, the allene groups of aromatic rings were observed at 2070 cm^−1^ for FHC and at 2100 cm^−1^ for FAC, which have been shifted to 2120 cm^−1^ and 2110 cm^−1^, respectively, indicating the involvement of π−π interactions in the adsorption of MB [48,49,50].

**Figure 8:**
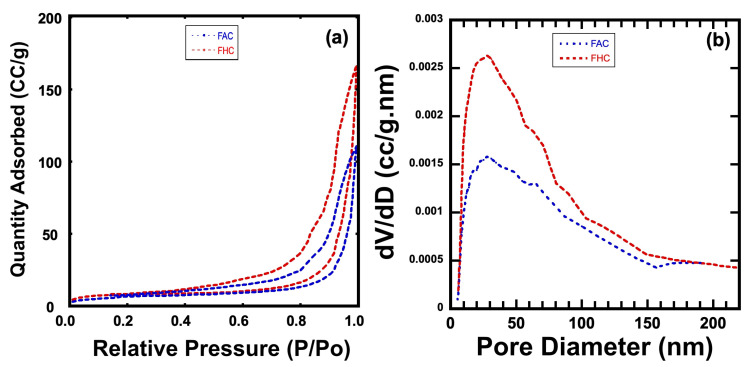
(**a**)The N_2_ adsorption-desorption isotherms of FHC and FAC and (**b**) the pore size distribution of FHC and FAC.

Overall, comparing the Langmuir and Freundlich fitting values of the two models (Table 1) shows that the FHC sample is more effective in adsorbing MB dyes than the FAC sample. It appears that the chemical triggering by ZnCl_2_ in the synthesis of the FAC resulted in extensive hydrolysis, which may lead to aggregations and pore blockage. Given that the chemical activation led to a decline in the anticipated features, it appears that MHC is an effective method and does not require further activation. This is supported by the N_2_ isotherm results presented in Figure 8. The isotherm exhibits a type-IV isotherm with a H_3_ hysteresis, which represents a broader pore size range [51,52]. The lack of capillary condensation closure denotes that those pores sizes are broadly distributed and that there are macropores [52,53,54,55]. The FHC sample shows a higher nitrogen gas uptake, of—170 cm^3^/g—than the FAC sample, with a gas uptake of ~100 cm^3^/g. The pore size distribution of both samples covers a range of 5–220 nm, with a maximum of around 28 nm. For the FHC and FAC samples, the surface areas are 25.8 m^2^/g and 22.6 m^2^/g, and the pore volumes are 0.258 cm^3^/g and 0.149 cm^3^/g, respectively.

The prepared composites show potential in the adsorption of MB dye when compared with the recent literature, as shown in Table 3. The FHC and FAC prepared in the current research have a removal percentage (83.9–87.5%) and a maximum methylene blue adsorption capacity of 556 mg/g and 50 mg/g, respectively. X. Zhang et al. treated Chinese medicine industry waste using the hydrothermal carbonization method, followed by alkali activation. Their samples had a surface area in the range of 42–53 m^2^/g and had a removal percentage for MB dye in the range of 60–80% [36]. Y. Haung et al. prepared MgAl-hydrochar nanocomposites using the hydrothermal method and found that they had a maximal adsorption capacity of Langmuir (*q_L_*) for MB dye of 257 mg/g [56]. T. Tran et al. used coffee husk to prepare hydrochar with a surface area of 34 m^2^/g, which was used in the adsorption of MB dye with a maximum methylene blue adsorption capacity of 104 mg/g [57]. G. Davies et al. used avocado seeds to prepare hydrochar and iron oxide-carbon through hydrothermal carbonization. They applied the prepared samples in MB dye adsorption and found their maximum adsorption capacity to be about 246 mg/g and 25 mg/g, respectively [17]. In this work, iron oxide-hydrochar composite and iron oxide-activated hydrochar were synthesized through microwave hydrothermal carbonization treatment. This work offers a simple synthesis procedure as the iron oxide was synthesized simultaneously with hydrochar in the same autoclave, saving time and effort. Using a microwave instead of conventional heating saves time and energy. Microwave-assisted hydrothermal carbonization treatment will be applied to different food wastes to explore its potential for producing efficient adsorbents for different pollutants.

## 3. Materials and Methods

The succeeding substances—ferrous chloride tetrahydrate, Methylene Blue dye, anhydrous zinc chloride, potassium hydroxide, sodium hydroxide, ammonia (25% NH_3_), hydrochloric acid (37%), sodium chloride, and ferric chloride hexahydrate—were used as delivered, without any further treatment. Solutions were prepared using D.I. water.

The tunning of the pH was followed up by a pH meter, Orion 2 Star, from Thermos-Fisher Scientific (Waltham, MA, USA). The FT-IR analysis was conducted on a Cary 630 FT-IR spectrophotometer. The Bruker D8 X-ray Diffractometer was used for the XRD study, which produced X-rays with a wavelength of 0.15418 nm at 35 × 10^3^ V and 25 × 10^−3^ A, utilizing glancing angles from 10 to 60 at scanning increments of 0.02 and with an accuracy of 0.001. Using a SEM (Philips XL30, Hillsboro, OR, USA) with an accelerating voltage of 30 × 10^3^ V and a magnification of up to 400.000, the surface morphology was examined. TEM imaging was performed using a JEOL JEM-1011 Transmission Electron Microscope with high resolution. A UV-1800 spectrophotometer (Shimadzu, Kyoto, Japan) was used to follow the adsorption of MB. Using a BET surface area analyzer (Micromeritics, ASAP 2020, Norcross, GA, USA), the N_2_ adsorption was carried out at 77 K. For 5 h, the composites were degassed at 300 °C to clean their surface. The BJH method was adapted to calculate the pore size distribution from the adsorption branch.

### 3.1. Preparation of Iron Oxide (F)

Iron oxide was synthesized following our previous work [61]. A mixture of FeCl_2_•4H_2_O and FeCl_3_•6H_2_O with a 1:2 molar ratio was prepared. Ammonia was added gradually until the pH reached 10 while under continuous magnetic stirring. The solution was left under stirring for 2 h and at a temperature of 60 °C. This is used later as the iron oxide precursor (FP). For comparison purposes, part of the FP was subjected to the same microwave-assisted hydrothermal treatment through heating at 200 °C for 1 h. First, this sample was washed, then dried at 80 °C for 12 h and named (F).

### 3.2. Preparation of Iron Oxide-Hydrochar Composite (FHC)

**Scheme 1:**
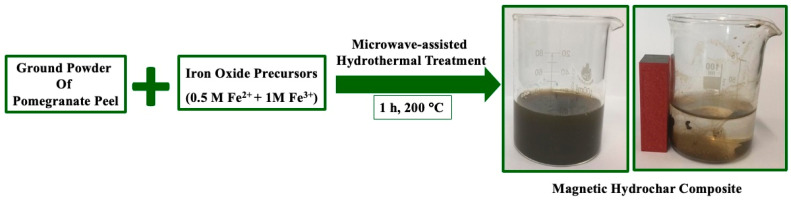
Iron oxide-hydrochar composite (FHC) prepared by MHC treatment of pomegranate peels’ waste.

Pomegranate peels were cleaned with tape water then D.I. water, dried and ground, then subjected to a basic treatment. Then, they were named basic pomegranate peels (BPP), as discussed in our previous publication [6]. A mixture of 6 g of BPP, 1 g of prepared iron oxide precursor, and 60 mL D.I. water was left under continuous magnetic stirring for 1 h at room temperature. The mixture was transferred to a Teflon autoclave, which was then heated for 1 h at 200 °C in a microwave. A scheme representing the synthesis steps is shown in Scheme 1. First, this sample was washed, then dried at 80 °C for 12 h and named FHC.

### 3.3. Preparation of Iron Oxide-Activated Hydrochar Composite (FAC)

A mixture of 6 g of BPP, 1 g of prepared iron oxide precursor, 10 mL of 6 M ZnCl_2,_ and 50 mL D.I. water was left under continuous magnetic stirring for 1 h at room temperature. The mixture was transferred to a Teflon autoclave, which was then heated for 1 h at 200 °C in a microwave. First, this sample was washed, then dried at 80 °C for 12 h and named FAC. 

### 3.4. Determining the pH of Point of Zero Charge (pH_pzc_)

A mixture of 0.2 L of 0.1 M NaCl solution and 0.2 g of FHC or FAC was stirred at room temperature for 1 day to establish the equilibrium, then the equilibrium pH (pH_e_) was measured. The NaCl solutions have different pH values, which were altered by 0.1 M NaOH or 0.1 M HNO_3_ to cover a range of initial pH (pH_i_) from 2 to 12.

### 3.5. Adsorption Equilibrium of Methylene Blue Dye

Next, 100 ppm MB dye solution was prepared by mixing 0.1 g of MB dye and 1 L of DD water. This stock solution was consecutively diluted to prepare a series of MB dye solutions with starting concentration *C_i_* = 5, 10, and 50 ppm. By using 0.1 M NaOH and the pH meter, the pH was kept at 8.

A series of batch adsorption tests were conducted with each of the composites to perform the equilibrium analysis. Specifically, 50 mL of each MB solution, with its initial concentration (*C_i_*) in the range of 5–100 ppm, was combined with 0.1 g of the composite FHC or FAC. To ensure the mixture reached equilibrium, it was agitated continuously for 24 h at 25 °C and 350 rpm. The sample was then centrifuged, and the equilibrium concentration (*C_e_*) of the solution was determined using a UV-VIS spectrophotometer with a maximum wavelength of 664 nm. Equation (1) was used to compute the amount of MB dye adsorbed at equilibrium, while Equation (2) was used to calculate the removal efficiency of MB dye.

(1) $$q_{e}=\frac{\left(C_{i}-C_{e}\right)}{m}\;V\;$$

(2) $$R\%=\frac{\left(C_{i}-C_{e}\right)}{C_{i}}\times 100\;$$

where *q_e_* is the adsorption capacity at equilibrium (mg/g), *C_i_* and *C_e_* are the starting and the equilibrium concentrations (ppm), respectively. *V* stands for the volume of the solution in millimeters, *m* is the used mass of adsorbent in g, and *R*% is the removal efficiency.

Langmuir and Freundlich adsorption isotherms were used to fit the experimental data through nonlinear regression with the aid of the CAVS—adsorption evaluation 2.0 program—using Equations (3) and (4), respectively.

(3) $$q_{e}=q_{l}*K_{l}*C_{e}/\left(1+K_{l}*C_{e}\right)\;$$

(4) $$q_{e}=K_{fr}*\;{C_{e}}^{1/n_{fr}}$$

where *q_L_* is the Langmuir monolayer sorption capacity (mg/g), *K_L_* is Langmuir constant, which reveals how much the adsorbate and adsorbent interact, *K_fr_* is the Freundlich constant ((mg/g)(L/mg)), and *n_fr_* is the Freundlich exponent.

Equation (5) can be used to obtain *R_L_*, a separation factor or equilibrium parameter from the Langmuir isotherm.

*R_L_* = 1/(1 + *K_L_* × *C_i_*) (5)

when its value is between 0–1, it is a favorable adsorption.

Moreover, the Dubinin–Radushkevich (D–R) model is used to describe the nature of the adsorption process as a chemical or a physical adsorption, and it is represented by the following linear equation:

(6) $$lnQ_{e\;}=lnQ_{m\;\;}-\beta \varepsilon^{2}\;$$

where β is a constant related to the mean free energy of adsorption (mol^2^ /KJ^2^) and ε is the Polanyi potential (KJ/mol), and is calculated according to the following equation:

(7) $$\varepsilon =RT\ln(1+\frac{1}{C_{e}})\;$$

where *R* is the gas constant, *T* is the temperature in Kelvin, and *C_e_* is the equilibrium concentration [43,44]. The mean adsorption energy (*E*) is calculated as following:

(8) $$E=\frac{1}{\sqrt{2\beta }}$$

## 4. Conclusions

In this work, microwave-assisted hydrothermal carbonization treatment (MHC), a time- and energy-efficient method, was successful in the synthesis of an iron oxide-hydrochar composite and iron oxide-activated hydrochar under the effect of the chemical activation by ZnCl_2_. As has been proven by the various characterization techniques, amorphous carbon and crystalline iron oxide were formed. The hydrochar in the FHC and FAC had a porous structure, and the iron oxide had a rod-like structure. The adsorption performance for the removal of MB dye was promising, as shown by the FHC sample with a maximum adsorption capacity of 556 mg/g achieved in this work. Furthermore, it is vital for the adsorbent to have a high sensitivity at a low concentration of pollutants for environmental applications. More than 85% removal of the MB dye from 5 ppm was achieved by the FHC sample. Therefore, in terms of being environmentally friendly, safe, straightforward, and affordable, this time- and energy-efficient technology may compete with other approaches to producing highly effective adsorbents. The microwave-assisted hydrothermal carbonization treatment can be applied with different agricultural wastes to produce hydrochar, which can be used as an adsorbent for various pollutants in future work.

## Figures and Tables

**Table 1:** Adsorption isotherm parameters for MB dye by FHC and FAC samples.

	Langmuir			Freundlich			Dubnin–Radushkevich		
	*q_L_*	*K_L_*	R^2^	*K_f_*	*n_fr_*	R^2^	*Q_m_*	E	R^2^
FHC	556.33	0.005	0.999	2.988	1.059	0.999	24.05	10.31	0.833
FAC	50.418	0.281	0.968	11.273	1.781	0.93	30.88	12.27	0.973

**Table 2:** Separation factors (*R_L_*) for samples FHC and FAC at different starting concentrations of MB dye.

*C_i_* (ppm)	5	10	50	100	Average *R_L_*
FHC	0.9758	0.9527	0.8011	0.6681	0.8615
FAC	0.4155	0.2622	0.0664	0.03432	0.1858

**Table 3:** Comparing MB adsorption capacity in recent literature.

Adsorbents	Synthesis Method	Adsorption	Ref.
Hydrochar	Hydrothermal carbonization	R% = 60–80	[36]
Hydrochar-MgAl double hydroxide	Hydrothermal route	*q_L_* = 157 mg/g	[56]
Activated hydrochar	Hydrothermal carbonization	*q_L_* = 357 mg/g	[50]
Hydrochar	Hydrothermal carbonization	*q_L_* = 5.12 mg/g	[58]
Magnetic biochar	Microwave pyrolysis	*q_L_* = 144.9 mg/g	[59]
Activated Hydrochar	Hydrothermal carbonization	*q_L_* = 200.01 mg/g	[60]

*q_L_* is maximum Langmuir removal capacities, R% is the removal efficiency.

## Data Availability

The data are available upon request.
